# The Book World of Medicine and Science

**Published:** 1894-10-06

**Authors:** 


### The Book World of Medicine and Science.

Contemporary Science Series—"Man and Woman." Havelock Ellis. (London: Walter Scott. 1894.)

Not only is this latest addition to the Contemporary Series of a definitely instructive character, but it is a book possessing a special interest. Comparison in sex has never been more thoroughly and exhaustingly entered into than by our present authority, and Mr. Ellis has shown himself to have gained complete mastery of his subject. His authorities are widespread, and he has brought a vast amount of specialised information to support conclusions, many of which we are in sympathy with. But if we take up Mr. Ellis' book in the hope of finding that one sex is indisputably superior to the other, we shall be disappointed—differences there are, and differences there always must be. How far these differences exclude woman from partaking on equal terms in man's vocations is still the question of the hour. "We have to recognise," in our author's words, "that our present knowledge of men and women cannot tell us what they might be, or what they ought to be, but what they actually are under the condition of civilisation. By showing us that under varying conditions men and women are within certain limits indefinitely modifiable, a precise knowledge of the actual facts of the life of men and women forbids us to dogmatise rigidly concerning the respective spheres. . . . The small group of women who wish to prove the absolute inferiority of the male sex, the larger group of men who wish to circumscribe the sphere of women, must alike be ruled out of court." Later on, in concluding his most interesting work, Mr. Havelock Ellis says, "Our investigation shows us in what state of mind we ought to approach the whole problem—it is something to have asked the right question and to be set on the right road."

Gout and its Relation to Diseases of the Liver and Kidneys. By Robson Roose, M.D. 7th Edition. (London: H. K. Lewis, 1894.)

That there should be sufficient demand for this little work to warrant the issue of this the 7th edition, clearly indicates that the book must possess merits which appeal to a large class of readers. For our own part our admitted ignorance as to the essential pathology of the condition is in no way dissipated by the perusal of the 200 pages in which Dr. Roose synthesises the observations of authorities on the subject. The chemical aspect of gout if not altogether neglected, is not illumined by those brilliant flashes of light which the researches of Roberts and other chemists have thrown upon this obscure subject, and the author has altogether failed to render a clinical picture of gout in any of its heterogeneous manifestations. His chapters, however, on the treatment of gout and its various complications are to our mind by far the most valuable sections of the work, show considerable practical common sense, and afford many useful hints both as to the handling of the general condition and the special treatment of complications. In this section the author refers to the use of piperazine as a means of removing uric acid from the system, he gives no particulars, but states that his results were not satisfactory. He, however, makes the statement that piperazine has no solvent action on uric acid in the presence of urine. This is altogether at variance with the results obtained by Dr. Gordon (*Brit. Med. Journ.*, June 15th, 1894), and hardly affects the question as to the solubility of uric acid in the presence of blood and tissue juices. The additions which this edition embraces are useful and to the point, and render the book as a treatise on gout far more valuable and complete than the former editions.

